# Percentage of body fat is associated with increased risk of diverticulosis: A cross sectional study

**DOI:** 10.1371/journal.pone.0264746

**Published:** 2022-03-01

**Authors:** Chi-Wei Shih, Yu-Hsin Chen, Wei-Liang Chen

**Affiliations:** 1 Department of Internal Medicine, Tri-Service General Hospital, School of Medicine, National Defense Medical Center, Taipei, Taiwan, Republic of China; 2 Department of Emergency Medicine, Hualien Armed Forces General Hospital, Tri-Service General Hospital, National Defense Medical Center, Taipei, Taiwan, Republic of China; 3 Department of Emergency Medicine, Taichung Veterans General Hospital, Taichung, Taiwan, Republic of China; 4 Division of Family Medicine, Department of Family and Community Medicine, Tri-Service General Hospital, School of Medicine, National Defense Medical Center, Taipei, Taiwan, Republic of China; 5 Division of Geriatric Medicine, Department of Family and Community Medicine, Tri-Service General Hospital; and School of Medicine, National Defense Medical Center, Taipei, Taiwan, Republic of China; 6 Department of Biochemistry, National Defense Medical Center, Taiwan, Republic of China; School of Digestive & Liver Diseases, Institute of Post Graduate Medical Education & Research, INDIA

## Abstract

**Background:**

Obesity has been indicated to be a risk factor of diverticulosis. However, plausible relationship remained controversial. This cross-sectional study elucidated the association between percentage of body fat and the risk of diverticulosis.

**Methods:**

The study was conducted at a single medical center in Taiwan from 2000–2016 which enrolled 5557 adults with age above 20 years old receiving a health examination including self-reported questionnaires, measurement of percentage of body fat (PBF), blood test and colonoscopy at the Tri-Service General Hospital (TSGH). Logistic regressions were used to analyze the association between PBF and diverticulosis. Further stratification of participants was based on age and gender and three extended models were established for multivariable adjustment.

**Results:**

243 of 3141 males and 103 of 2416 females were diagnosed with having diverticulosis. After covariates adjustment, only participants in the highest quartile of PBF (Q4 ≥33.8%) showed significantly positive association with the risk diverticulosis (OR 2.089, p <0.001). In subgroup analysis, the odds ratio for having diverticulosis in females was significantly higher than in males. In addition, We found that the odds ratio of having diverticulosis was higher in the group older than 60 years old compared to the younger group (OR 1.052; p<0.001; OR 1.043; p<0.001).

**Conclusions:**

In conclusion, PBF was a potential risk factor of diverticulosis. Individuals with higher PBF exhibits increased risk of diverticulosis, especially in females. Furthermore, bioelectrical impedance analysis may create a simple, available and radiation-free way to assess the risk of diverticulosis.

## Introduction

Colonic diverticulosis is a condition of having one or more diverticula protruding from mucosa and submucosa of colon. The real prevalence of diverticulosis remains unclear but it increases with age which has been reported in previous studies [1,2]. Besides, the incidence of diverticulosis has increased in Asia over the past decades [3]. In the past studies, frequent occurrence of constipation was thought to be a major cause of diverticulosis due to increased intraluminal pressure in colon. However, the hypothesis has been questioned by recent studies [4,5]. Most of patients with diverticulosis are asymptomatic. About 15% to 20% patients with diverticulosis develop clinical illness [6]. Three fourth of the symptomatic cases are presented with colicky abdominal pain without inflammation. The rest of one fourth develop diverticular bleeding, diverticulitis and a small number will complicate with bowel obstruction or abscess formation. The prevalence of diverticulosis is higher in old age than in young age, which is thought to be the most effective risk factor [7]. Several well-known risk factors of diverticulosis have also been reported in previous studies such as low dietary fiber, high total fat intake, physical inactivity and obesity [8–10]. As obesity is considered having a significant association with diverticulosis, many studies have been conducted with different methods of defining obesity. A retrospective study of Korean patients, Song et al. identified that there was no statistical significance between obesity and diverticulosis by multivariate analysis. [11]. In spite of Song’s study, a prospective cohort study conducted by Nagata reported that obesity defined by measuring visceral adipose tissue area had more sensitive correlation of diverticulosis than BMI [12]. Other parameters to define obesity, such as waist circumference and dietary habits have also been used in previous studies [13,14]. Furthermore, recent studies revealed that abdominal visceral fat measured by computed tomography provided a more predictive ability of diverticulosis than other parameters [12,15]. In our study, we measured percentage of total body fat to analyze the correlation between obesity and the incidence of diverticulosis. The aim of our study was to elucidate the relationship between the risk of diverticulosis and percentage of body fat by using bioelectrical impedance analysis.

## Material and methods

### Study sample

This cross-sectional study enrolled 5557 adults with age above 20 years old who underwent health examination at the TSGH from 2000–2016. The health examination included a laboratory data, body composition and detailed self-reported questionnaires. In addition, a diagnostic colonoscopy as a part of health examination was performed to identify any malignant polyps or other disease of colon such as diverticulosis. Before a diagnostic colonoscopy, all the participants were asked to complete detailed self-reported questionnaires. Those who reported any related abdominal symptom, such as abdominal tenderness or cramps, active rectal bleeding, fever, and nausea were not be able to have colonoscopy. All the colonoscopy was performed by experienced specialists after the participants had proper bowel preparation and fasted overnight. Any findings of diverticular bleeding, diverticulitis, familial polyposis syndrome or malignant tumors were excluded. All protocols in this cross-section study were approved by the Institutional Review Board (IRB) of TSGH. The TSGH IRB waived the need to obtain individual informed consent because these data were analyzed anonymously.

### Measures

Bioelectrical impedance analysis (BIA) was a validated for assessing body composition analysis [16]. In comparison with other medical equipment, such as dual-energy X-ray absorptiometry, computerized tomography or magnetic resonance imaging, BIA was more maneuverable and less expensive and radiation exposure. We measured PBF by using BIA (InBody720, Biospace, Inc., Cerritos, CA, USA).

### Assessments of covariates

The information of participants such as age, gender, smoking status and alcohol consumption were obtained by a self-completed questionnaire. We acquired smoking status by asking the question “Do you have a smoking habit”. For the alcohol consumption, we divided participants into two groups which were alcohol consumption and those who never consumed alcohol. All the participants had been fasting for at least 8 hours prior to blood draw. Blood samples such as total cholesterol, triglycerides, creatinine, and glucose were analyzed by standard methods.

### Statistical analysis

Student’s t test was used to assess the differences in continuous variables, such as age, percentage of total body fat, serum cholesterol, triglycerides, creatinine, and fasting glucose. Smoking habit and alcohol consumption were regarded as categorical variables which were assessed by using Pearson’s chi-square tests. Three extended models were established for multivariable adjustment. The respective models were adjusted as follows: Model 1 = unadjusted; Model 2 = age, sex, total cholesterol, creatinine, triglyceride, fasting glucose; Model 3 = Model 2 + smoking, alcohol consumption. Logistic regression was applied to analyze the association between PBF and the risk of diverticulosis. Furthermore, we used receiving operator characteristic (ROC) curve analysis to determine the ideal gender-specific cut-off point for PBF to predict the risk of diverticulosis. We used the Youden’s index in order to find the ideal cut-off point which was regarded as the shortest distance between the upper left corner of the graph and the ROC curve. Two-side p values of less than or equal to 0.05 were considered as significant difference. All statistical analyses of this study were analyzed between June and August 2019 by using the Statistical Package for the Social Sciences, version18.0 (SPSS Inc., Chicago, IL, USA).

## Results

5557 participants with 3141 males and 2416 females were enrolled in the study. Table 1 disclosed baseline characteristics of the study group. In our study, the females were prone to having higher percentage of body fat and total cholesterol and lower serum creatinine, triglyceride and fasting sugar. Smoking history and alcohol consumption were more prominent among males.

**Table 1:** Characteristics of study participants.

	Male (n = 3141)	Female (n = 2416)	*P* Value
**Continuous variables** ^**a**^ **, mean (SD)**			
**Percentage of body fat**	025.80 (6.20)	032.89 (6.61)	<0.001
**Age (years)**	051.67 (12.33)	050.51 (11.81)	<0.001
**Serum TC (mg/dL)**	191.35 (36.81)	196.16 (37.06)	<0.001
**Serum Creatinine (mg/dL)**	000.97 (0.32)	000.68 (0.20)	<0.001
**Serum TG (mg/dL)**	158.60 (99.34)	115.93 (64.63)	<0.001
**Serum FG (mg/dL)**	100.09 (29.61)	094.32 (21.56)	<0.001
**Categorical variables** ^**b**^ **, n (%)**			
**Smoking**	052.0	011.1	<0.001
**Alcohol drinking**	065.8	029.2	<0.001

Serum TC, serum total cholesterol; Serum TG, serum triglyceride; Serum FG, serum fasting glucose.

^a^ Values were expressed as mean (standard deviation).

^b^ Values in the categorical variables were expressed as number (%).

Table 2 showed the associations between diverticulosis and percentage of body fat. Among all participants, the odds ratio for having diverticulosis in unadjusted model 1 was 1.030 (95% CI 1.015–1.045, *p*<0.001). In adjusted model 2 and fully adjusted mode 3, the odds ratio were 1.045 (95% CI 1.025–1.064, *p*<0.001) and 1.045 (95% CI 1.025–1.065, *p*<0.001) which did not affect the statistical significance. We further divided the study population into two groups by gender in Table 2. In the fully adjusted model 3, the odds ratio for having diverticulosis in females was significantly higher than in males (odds ratio 1.076, *p* <0.001; odds ratio 1.029, *p* = 0.001, respectively).

**Table 2:** Risks of diverticulosis between percentage of body fat and genders.

	Total (n = 5557)		Male (n = 3141)		Female (n = 2416)	
	OR(95% CI)	*P* value	OR(95% CI)	*P* value	OR(95% CI)	*P* value
**Model 1**	1.030 (1.015, 1.045)	<0.001	1.045 (1.024, 1.067)	<0.001	1.117 (1.083, 1.152)	<0.001
**Model 2**	1.045 (1.025, 1.064)	<0.001	1.029 (1.005, 1.052)	<0.001	1.076 (1.039, 1.114)	<0.001
**Model 3**	1.045 (1.025, 1.065)	<0.001	1.029 (1.006, 1.053)	<0.001	1.076 (1.039, 1.114)	<0.001

Adjusted covariates

Model 1 = unadjusted.

Model 2 = Age, sex, total cholesterol, creatinine, triglyceride, fasting glucose.

Model 3 = Model 2 + smoking, alcohol consumption.

Additionally, the participants were divided into two groups by age as shown in Table 3. Among the participants older than 60 years old, the odds ratios was 1.052 (*p*<0.001) for both mode1 2 and fully adjusted model 3 which were respectively significantly higher than the participants younger than 60 years old for having diverticulosis (odds ratio 1.042, *p* <0.001; odds ratio 1.043, *p* = 0.001, respectively). Moreover, we applied ROC curve to decide an optimal gender-specific cut-off point for percentage of body fat to predict the risk of diverticulosis. The results were shown in Table 4 and presented the significant associations with the risk of diverticulosis in each of the models of the two genders.

**Table 3:** Risks of diverticulosis between percentage of body fat and age.

	Total (n = 5557)		Age<60 (n = 4195)		Age>60(n = 1362)	
	OR(95% CI)	*P* value	OR(95% CI)	*P* value	OR(95% CI)	*P* value
**Model 1**	1.030 (1.015, 1.045)	<0.001	1.021 (1.001, 1.041)	0.039	1.028 (1.004, 1.052)	0.019
**Model 2**	1.045 (1.025, 1.064)	<0.001	1.042 (1.017, 1.068)	0.001	1.052 (1.021, 1.085)	0.001
**Model 3**	1.045 (1.025, 1.065)	<0.001	1.043 (1.018, 1.069)	0.001	1.052 (1.020, 1.085)	0.001

Adjusted covariates

Model 1 = unadjusted.

Model 2 = Age, sex, total cholesterol, creatinine, triglyceride, fasting glucose.

Model 3 = Model 2 + smoking, alcohol consumption.

**Table 4:** Risks of diverticulosis between percentage of body fat and genders (according to gender-specific cut-off point).

**Men(n = 3141)**	**OR(95% CI)**	**P value**
Model 1	1.877 (1.382, 2.550)	<0.001
Model 2	1.514 (1.101, 2.081)	0.011
Model 3	1.514 (1.101, .083)	0.011
**Women(n = 2416)**		
Model 1	3.936 (2.576, 6.006)	<0.001
Model 2	2.550 (1.628, 3.994)	<0.001
Model 3	2.548 (1.625, 3.993)	<0.001

Adjusted covariates

Model 1 = unadjusted.

Model 2 = Age, sex, total cholesterol, creatinine, triglyceride, fasting glucose.

Model 3 = Model 2 + smoking, alcohol consumption.

In Table 5, the participants were further divided into quartiles by PBF (Q1 < 24%; Q2: 24% to< 28.5%; Q3: 28.5% to< 33.8%; and Q4 ≥33.8%). We further displayed the association between the quartiles of PBF and the risk of diverticulosis. With fully adjustment of covariates, the odds ratios for diverticulosis among subjects with different PBF quartile were Q2: 1.304 (*p* = 0.117), Q3: 1.163 (*p* = 0.405) and Q4: 2.089 (*p*<0.001). Only participants in the highest quartile of PBF (Q4 ≥33.8%) showed significantly positive association with the risk diverticulosis (odds ratio: 2.089, p<0.001) after fully multivariate adjustments. In addition, the data were further analyzed separately by gender, as shown in Table 6. In Model 3, which was fully adjusted by multivariable analysis, there was a significant positive association between the highest quartile of PBF (Q4 ≥37.1%) and risk of diverticulosis in the female group with odds ratio of 2.522 (*p* = 0.014).

**Table 5:** Risks of diverticulosis by quartile of percentage of body fat.

PBF (%)	Model 1	P value	Model 2	P value	Model 3	P value
Quartile 1 (< 24)	1.0 (reference)		1.0 (reference)		1.0 (reference)	
Quartile 2 (24–28.5)	1.421 (1.029, 1.962)	0.033	1.307 (0.938, 1.820)	0.114	1.304 (0.936, 1.816)	0.117
Quartile 3 (28.5–33.8)	1.137 (0.810, 1.594)	0.458	1.163 (0.815, 1.659)	0.406	1.163 (0.815, 1.660)	0.405
Quartile 4 (≥ 33.8)	1.688 (1.234, 2.309)	0.001	2.089 (1.439, 3.034)	<0.001	2.089 (1.436, 3.030)	<0.001

Adjusted covariates

Model 1 = unadjusted.

Model 2 = Age, sex, total cholesterol, creatinine, triglyceride, fasting glucose.

Model 3 = Model 2 + smoking, alcohol consumption.

**Table 6:** Risks of diverticulosis between percentage of body fat and genders (by quartile of percentage of body fat).

**PBF of Male (%)**	**Model 1**	**P value**	**Model 2**	**P value**	**Model 3**	**P value**
Quartile 1 (< 21.8)	1.0 (reference)		1.0 (reference)		1.0 (reference)	
Quartile 2 (21.8–25.8)	1.462 (0.967, 2.209)	0.072	1.184 (0.777, 1.805)	0.431	1.185 (0.777, 1.806)	0.431
Quartile 3 (25.8–29.7)	1.647 (1.101, 2.465)	0.015	1.247 (0.822, 1.891)	0.299	1.248 (0.822, 1.894)	0.298
Quartile 4 (≥ 29.7)	2.025 (1.368, 2.996)	<0.001	1.479 (0.979, 2.233)	0.063	1.488 (0.984, 2.248)	0.059
**PBF of Female (%)**	**Model 1**	**P value**	**Model 2**	**P value**	**Model 3**	**P value**
Quartile 1 (< 28.3)	1.0 (reference)		1.0 (reference)		1.0 (reference)	
Quartile 2 (28.3–32.9)	1.703 (0.773, 3.750)	0.186	1.265 (0.566, 2.831)	0.567	1.272 (0.568, 2.846)	0.559
Quartile 3 (32.9–37.1)	2.681 (1.281, 5.610)	0.009	1.533 (0.710, 3.310)	0.276	1.538 (0.713, 3.320)	0.272
Quartile 4 (≥ 37.1)	5.400 (2.712, 10.753)	<0.001	2.518 (1.203, 5.269)	0.014	2.522 (1.206, 5.277)	0.014

Adjusted covariates

Model 1 = unadjusted.

Model 2 = Age, sex, total cholesterol, creatinine, triglyceride, fasting glucose.

Model 3 = Model 2 + smoking, alcohol consumption.

## Discussions

### Main findings

The present study showed a positive correlation between obesity and diverticulosis. In our analysis, participants with higher PBF were more likely to have diverticulosis. In addition, potential confounders were adjusted, such as age, gender, total cholesterol, triglyceride, creatinine, fasting glucose, alcohol consumption and smoking status. We found that females with higher PBF tended to have higher risk of diverticulosis in univariable analysis and the positive association was not displayed in males. Furthermore, we found that there was a stronger predictive ability for diverticulosis with a use of ROC curve analysis to decide an optimal gender-specific cut-off point for PBF.

### Comparison with other studies

Few previous studies examined the impacts of BMI-defined obesity on diverticulosis and none of them revealed positive associations [11,17,18]. In contrast, a meta-analysis included 9 cross-sectional studies and 1 prospective cohort study through January 2017 reported an association between BMI-defined obesity and the risk of diverticulosis [19]. In spite of the positive relationship, the statistical heterogeneity of the meta-analysis was high and each of the studies from eastern countries revealed no association between obesity and diverticulosis which were opposite to the results of western countries. Two recent studies focused on the association with body fat, especially visceral body fat, on the risk of diverticulosis [12,15]. One study by Lee showed that total abdominal fat, abdominal visceral fat, and abdominal subcutaneous fat were significantly higher in the diverticulosis subgroup than in the controls. Besides, the other study by Nagata showed that diverticulosis was significantly associated with visceral adipose tissue area, even in people with normal weight [12]. The body fat of the two studies was both measured by CT scan.

### Strengths

In our studies, bioelectrical impedance analysis was used as the measurement of PBF which created a non-invasive, maneuverable and radiation-free method to assess the risk of diverticulosis. Although the mechanism that linked obesity to diverticulosis still remained unclear, several connections behind the association had been promoted by previous studies. It was suggested that increasing intraluminal pressure was a plausible cause of diverticulosis. The speculated mechanism was intraluminal pressure caused diverticula protruding from mucosa and submucosa of colon. Besides, several studies identified obese individuals produced higher concentration of methane gas by gut bacterial flora than nonobese individuals. A higher concentration of methane gas in gut might lead to delaying transition of small bowel and decreasing motility of colon which was conjectured to increase intraluminal pressure and resulted in diverticulosis.

Besides, the correlation between gender and diverticulosis remained controversial in previous studies [17,20,21]. In a retrospective study of Japanese patients, Yamamichi et al. identified there was a significant positive correlation between diverticulosis and male subgroups in multivariate analysis [10]. However, in a prospective study of Korean patients, Song et al. reported no association between gender and diverticulosis [11]. We also found that the participants with older age were more likely to have diverticulosis than the younger participants which was in line with previous studies in Taiwan [20,21].

### Limitations

Our study had four limitations needed to be considered. First, a few well-known lifestyles factors were not taken into account, such as dietary fiber intake and physical activity. However, the correlation between fiber intake and diverticulosis was still unclear. Several studies reported that low dietary fiber was associated with diverticulosis, but others revealed conflicting data [22–24]. Physical activity was shown to have an inversely correlation with the risk of symptomatic diverticular disease in a large prospective study [9]. Despite that, to our knowledge, the association between dietary, physical activity and PBF should be existed. Thus, investigations of dietary and physical activity should be involved in the questionnaire in future studies. Second, it was well known that multiple risk factors were associated with diverticulosis. In our study, age, gender and PBF might be important risk factors in diverticulosis. However, the other risk factors needed to be considered as predictors and might have varying degrees of effects on diverticulosis. Third, self-election bias of the participants in the study might exist because all enrolments received health examination at their own expense, which meant that they intended to pay more attention to their health condition and higher economic status among population in Taiwan. Finally, we acquired smoking status by asking the question “Do you have a smoking habit”. For the alcohol consumption, we divided participants into two groups which were alcohol consumption and those who never consumed alcohol. However, without having information about duration and daily amount of alcohol consumption as well as smoking status might lower the power of these factors. And forth, the proportion of diverticulosis in our research was relatively lower than other researches. This might be due to the selection bias we mentioned above, and moreover, the sensitivity of colonoscopy to detect diverticulosis was less than radiological techniques [25].

## Conclusions

In conclusion, PBF was a potential risk factor of diverticulosis. Individuals with higher PBF exhibits increased risk of diverticulosis, especially in females. PBF measured by bioelectrical impedance analysis may be a simple, available and radiation-free tool for assessing the risk of diverticulosis. Further longitudinal surveys and interventional designs are necessary to confirm the conclusions.
